# Risk factors for neurological complications in adult ECMO patients: a systematic review and meta-analysis

**DOI:** 10.3389/fmed.2025.1726621

**Published:** 2026-02-10

**Authors:** Meijun Dong, Peipei Gu, Lingyun Cai, Yan Zhu, Li Sheng, Jiangshuyuan Liang, Shuhua Zhao, Fei Zeng

**Affiliations:** Department of Nursing, The Second Affiliated Hospital of Zhejiang University School of Medicine, Hangzhou, China

**Keywords:** extracorporeal membrane oxygenation, intracranial hemorrhage, neurological complications, risk factors, stroke, systematic review

## Abstract

**Objective:**

Aiming to systematically review risk factors for neurological complications in adults receiving ECMO support.

**Methods:**

A comprehensive computerized search was conducted in Chinese and English databases for studies examining risk factors of neurological complications in adult ECMO patients, supplemented by manual and reference tracking, with the search period extending up to April 2025. Meta-analysis and sensitivity analyses were performed using Stata 18.0 software. 29 studies involving 62,656 patients were included. The meta-analysis results showed that female sex, pre-ECMO cardiac arrest, renal replacement therapy, and cardiac insufficiency were influencing factors of composite neurological complications in adult ECMO patients. Female sex and ECMO duration were risk factors of stroke. Female sex, pre-ECMO lactate, pre-ECMO pH, platelet count, low platelets, APTT, and vasoactive drug use were risk factors of intracranial hemorrhage. The pooled results for female sex in the stroke group was not robust. Significant publication bias was observed for composite neurological complications outcomes, whereas no significant publication bias was detected for stroke or intracranial hemorrhage.

**Conclusion:**

Given the diverse etiologies underlying neurological injury in adult ECMO recipients, protocolized neurological monitoring and prompt intervention for detected abnormalities are strongly recommended.

**Systematic review registration:**

PROSPERO (CRD420251069285).

## 1 Introduction

Extracorporeal membrane oxygenation (ECMO) is an advanced life support technology that utilizes an extracorporeal circulation system to provide prolonged cardiopulmonary support, including gas exchange and systemic circulatory support (1). This technique is beneficial for sustaining the lives of critically ill patients, buying time for reversible pathological changes. It is also frequently employed as a bridging therapy for lung transplantation, facilitating organ function recovery or serving as a transitional measure prior to transplantation (2). The use of ECMO support in severe cardiopulmonary failure has advanced rapidly. However, neurological complications remain one of the most common complications during ECMO, including intracranial hemorrhage, ischemic stroke, seizures, hypoxic–ischemic brain injury, brain death, cerebral infarction, and cerebral edema (3). Studies report that the incidence of neurological complications during ECMO can be as high as 44% (4). Significant risk factors include hypercapnia, duration of ECMO support, serum lactate levels, platelet count, and anticoagulation therapy (5–8). Importantly, neurological complications constitute a major independent predictor of mortality in ECMO patients (9).

Although studies have investigated its incidence and influencing factors, systematic reviews of these risk factors remain limited, and the mechanisms underlying neurological complications are not yet fully understood. This study aims to systematically review and perform a meta-analysis of existing research on neurological complications in adult ECMO patients through comprehensive literature retrieval, thereby providing evidence-based references for the clinical management of neurological complications in ECMO patients.

## 2 Methods

Our study was developed according to the Preferred Reporting Items for Systematic Review and Meta-Analysis Protocols and registered on PROSPERO (CRD420251069285).

### 2.1 Literature search

**Figure 1:**
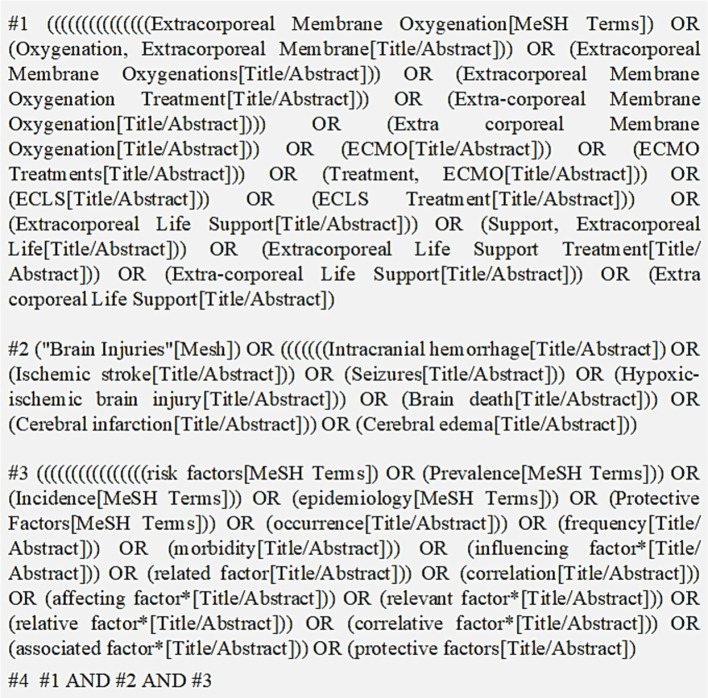
Search strategy (e.g., PubMed).

Comprehensive computerized searches were conducted in both Chinese and English databases, including China National Knowledge Infrastructure (CNKI), Wanfang Data, VIP, SinoMed, PubMed, Embase, Web of Science (WOS), Cochrane Library, and Cumulative Index to Nursing and Allied Health Literature (CINAHL). A search strategy combining subject headings with free-text terms was employed. Search terms such as extracorporeal membrane oxygenation, extracorporeal life support, ECMO treatments, ECMO, brain injuries, intracranial hemorrhage, ischemic stroke, seizures, hypoxic–ischemic brain injury, brain death, cerebral infarction, cerebral edema, risk factors, protective factors, influencing factor*, related factor, correlation, affecting factor*, relevant factor*, relative factor*, correlative factor*, and associated factor*. The search strategy (using PubMed as an example) is shown in Figure 1. Additionally, a manual review of references was conducted to supplement relevant literature. The search was limited to studies published up to April 2025.

Several studies originated from the Extracorporeal Life Support Organization (ELSO) registry and had overlapping enrollment periods. To avoid including duplicate patient cohorts, we compared study periods across all ELSO-derived publications and included only the study with the longest enrollment window, excluding the remaining smaller/overlapping studies.

### 2.2 Inclusion criteria and exclusion criteria

#### 2.2.1 Inclusion criteria

(1) Participants: Patients receiving extracorporeal membrane oxygenation (ECMO), aged > = 18 years; (2) Study Design: Case–control studies, cohort studies, or cross-sectional studies; (3) Outcome: Neurological complications newly developed during ECMO support or after weaning from ECMO. Neurological complications, objectively diagnosed as acute brain injury, intracranial hemorrhage, ischemic stroke, seizures, hypoxic–ischemic brain injury, brain death, cerebral infarction, or cerebral edema; (4) Published studies examining risk factors or predictive factors for neurological injury in adult ECMO patients; (5) Must provide multivariate analysis results including odds ratios (OR) with 95% confidence intervals (CI), or data convertible to OR, 95% CI, and standard error.

#### 2.2.2 Exclusion criteria

(1) Non-Chinese or non-English publications; (2) Unavailable or incomplete data; (3) Duplicate publications; (4) Gray literature (conference abstracts, posters, theses/dissertations, preprints, registry data, and non-peer-reviewed reports), case reports, expert consensus, or research with incomplete data; (5) Animal experiments or animal models;(6) Neurological complications or symptoms diagnosed before ECMO initiation.

### 2.3 Literature screening and data extraction

2 researchers independently screened the literature and extracted data according to the predefined inclusion and exclusion criteria. EndNote 20.0 software was used for deduplication and preliminary screening. Articles were initially selected based on title and abstract review, followed by full-text assessment and final inclusion determination based on quality evaluation. Cross-checking was performed for all included studies, with discrepancies resolved through group discussion or consultation with a third researcher. The extracted data included: author name(s), publication year, country, study design, study year, study population, outcome measures, Event count, Total population influencing factors and relevant data.

### 2.4 Literature quality assessment

If the study design was a case–control study or a cohort study, the Newcastle-Ottawa scale (NOS) was used, with scores of 0–3, 4–6, and 7–9 indicating low, moderate, and high quality, respectively. Only moderate- and high-quality studies were included in the meta-analysis, while low-quality studies were excluded.

### 2.5 Statistical analysis

Stata 18.0 software was used to analyze data. The odds ratio (OR) and 95% confidence interval (CI) for each variable were pooled as effect sizes, with statistical significance assessed using the Z-test (*p* < 0.05). Statistical heterogeneity among the included studies was assessed using Cochran’s Q test and the I^2^ statistic. The Q test was considered significant when *p* < 0.10, indicating the presence of heterogeneity. The I^2^ statistic quantified the proportion of total variation attributable to between-study heterogeneity, with values of approximately 25, 50, and 75% representing low, moderate, and high heterogeneity, respectively. When substantial heterogeneity (I^2^ > 50%) was detected, a random-effects model was applied; otherwise, a fixed-effect model was used. To assess the robustness of the results, sensitivity analysis was performed by comparing pooled estimates obtained using fixed-effect and random-effects models.

Furthermore, publication bias was evaluated for the most frequently studied influencing factor using funnel plots and Egger’s test. A statistically significant publication bias was considered present if *p* < 0.05, in which case the trim-and-fill method was employed to adjust the funnel plot. The significance level was set at *α* = 0.05 for all statistical tests.

Additionally, descriptive analysis was performed for influencing factors with an insufficient number of studies.

## 3 Results

### 3.1 Literature search results

**Figure 2:**
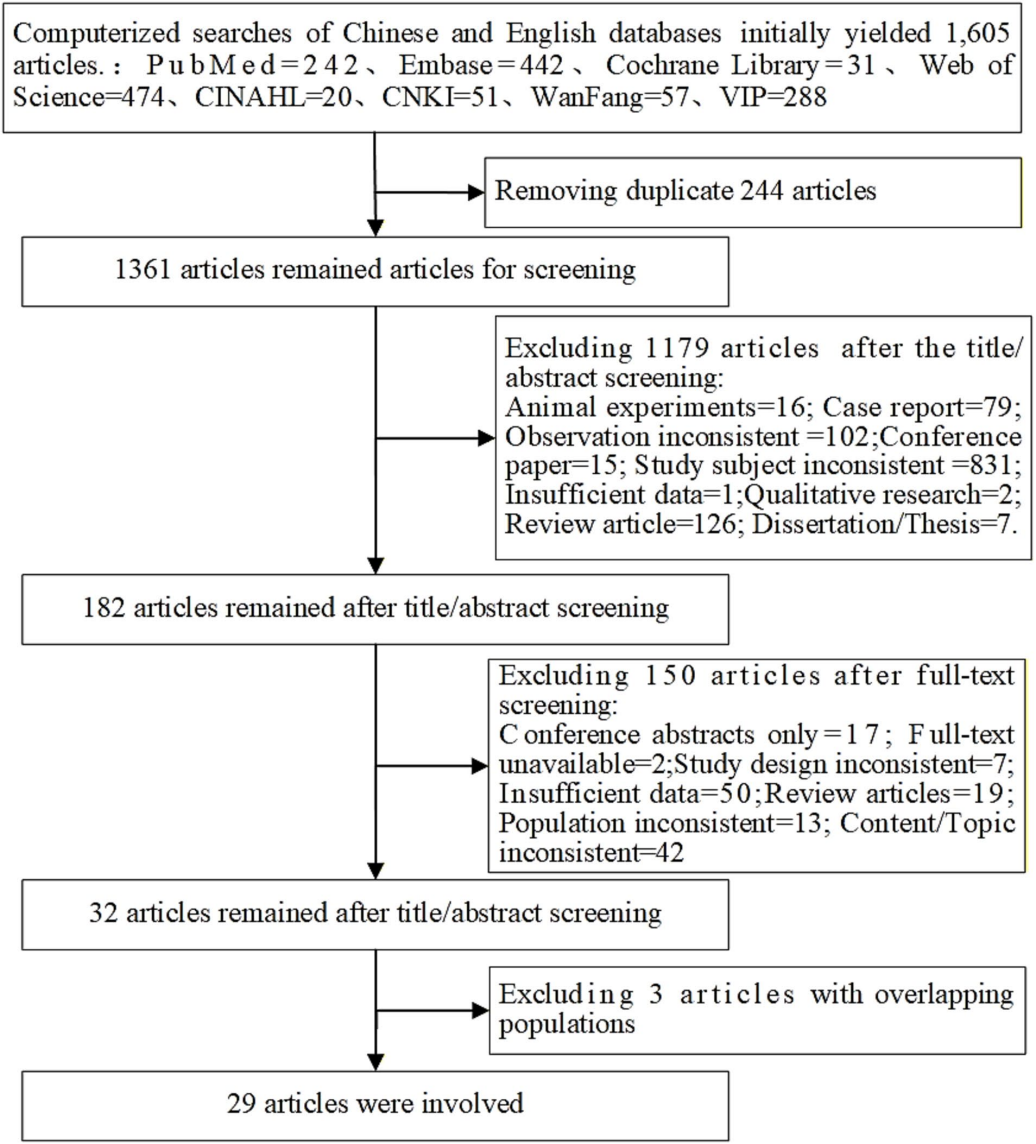
Literature search flowchart following the PRISMA guidelines.

A total of 1,605 relevant articles were identified through literature search. After removing duplicates, 1,361 records remained. Following preliminary screening, 186 studies were selected for full-text review. Based on quality assessment, 32 studies were included. Among these, 4 studies had overlapping study populations. We included only the study with the longest study period and excluded the other three, resulting in 29 studies being included in the final analysis. The specific process is shown in Figure 2.

### 3.2 Characteristics of included studies

Among the 29 included studies, 21 were cohort studies and 8 were case–control studies. A total of 62,656 patients were analyzed, among whom 6,087 were diagnosed with neurological complications. The studies spanned from 1992 to 2023, with individual sample sizes ranging from 37 to 20,297. A total of 39 influencing factors were identified. The basic characteristics of the included studies are presented in Table 1.

**Table 1:** Characteristics and quality assessment results of included studies.

Id	Study	Publication year	Country	Study design	Study years	Population	Outcome	Event count	Total population	Influencing factors	Quality assessment score
1	Almajed (31)	2024	America	case–control study	2018–2022	V-A ECMO	stroke	36	244	1–3	8
2	Balucani (32)	2024	America	cohort study	1992–2020	ECPR	neurological complications	1,286	7,460	4	8
3	Cavayas (33)	2020	Canada	cohort study	2012–2017	ECMO	neurological complications	832	11,972	5–8	6
4	Chapman (34)	2021	Australia	cohort study	2009–2017	ECMO	acute neurological complication	55	412	4, 7, 9	7
5	Hou (35)	2021	China	cohort study	2006–2016	V-A ECMO	neurological complications/intracranial hemorrhage	98	415	10, 11	7
6	Hunsicker (6)	2021	Germany	cohort study	2007–2018	V-V ECMO	intracranial hemorrhage	49	444	8, 12, 13	7
7	Huang (12)	2024	China	case–control study	2019–2023	ECMO	acute brain injury	118	584	5,14–17	8
8	Hwang (36)	2023	America	cohort study	2012–2021	V-A ECMO	ischemic stroke/ intracranial hemorrhage	983	20,297	18	7
9	Iacobelli (37)	2021	Sweden	cohort study	2010–2018	ECMO	cerebral infarction	41	275	9	8
10	Kasirajan (38)	1999	America	case–control study	1992–1996	V-A ECMO	intracranial hemorrhage	14	74	4, 19	8
11	Le Guennec (39)	2018	France	cohort study	2006–2014	V-A ECMO	ischemic stroke/ intracranial hemorrhage	65	878	4, 9, 19	7
12	Lorusso (17)	2016	Netherlands	cohort study	1992–2013	ECMO	neurological complications	682	4,522	1,5,20,21	6
13	Lüsebrink (7)	2022	Germany	cohort study	2016–2020	V-A ECMO	intracranial hemorrhage	70	598	12, 16, 22, 23	7
14	Luyt (8)	2016	France	cohort study	2006–2012	V-V ECMO	intracranial bleeding cerebral complications	18	135	8, 24	7
15	Malfertheiner (14)	2020	Germany	cohort study	2011–2016	V-A ECMO	intra-cranial ischemia and hemorrhage	32	187	18,20,25–29	9
16	Mateen (40)	2011	America	cohort study	2002–2010	ECMO	neurological diagnosis	42	87	24	6
17	Nunez (16)	2022	America	cohort study	2010–2017	V-V ECMO	ischemic stroke and intracranial hemorrhage	485	7,579	30–32	7
18	Omar (41)	2016	America	case–control study	2007–2014	ECMO	ischemic stroke	10	171	16	7
19	Ou (4)	2024	China	case–control study	2009–2021	V-A ECMO	acute brain injuries/ intracranial hemorrhage	66	150	12, 14	9
20	Pantel (42)	2022	Germany	cohort study	2011–2021	V-V ECMO	intracranial hemorrhage	43	204	33	7
21	Pozzebon (13)	2018	Belgium	cohort study	2008–2015	V-A ECMO	acute cerebral complication	18	56	34	7
22	Shi (10)	2023	China	case–control study	2015–2022	ECMO	neurologic impairment	24	37	35, 36	8
23	Shou (43)	2023	America	cohort study	2009–2020	ECMO	acute brain injury	488	3,125	18	6
24	Shou (2, 44)	2023	America	cohort study	2016–2021	V-A ECMO	acute brain injury	41	123	37	5
25	Wiest (45)	2024	Germany	cohort study	2006–2019	V-V ECMO	neurologic impairment	61	556	8, 12, 18	7
26	Wu (15)	2022	China	case–control study	2013–2019	V-V ECMO	neurologic impairment	11	77	22, 23	8
27	Zaaqoq (46)	2023	America	cohort study	2020–2021	V-V ECMO	stroke	43	595	32, 38	7
28	Toivonen (47)	2021	Sweden	case–control study	2010–2018	ECMO	neurologic injury	149	781	39	6
29	Yu (11)	2024	Germany	cohort study	2011–2021	ECMO	intracranial hemorrhage/ischemic stroke	227	618	12, 23, 33	6

Extracorporeal Membrane Oxygenation (ECMO); Veno-Arterial Extracorporeal Membrane Oxygenation (V-A ECMO); Veno-Venous Extracorporeal Membrane Oxygenation (V-V ECMO); Extracorporeal Cardiopulmonary Resuscitation (ECPR). 1 anticoagulation, 2 limb ischemia, 3 blood transfusion, 4 female, 5 pre-ECMO cardiac arrest, 6 hyperbilirubinemia, 7 Continuous renal replacement therapy(CRRT), 8 ΔPaCO2, 9 V-A ECMO, 10 low systolic blood pressure, 11 aortic vascular surgery combined with coronary artery bypass grafting, 12 platelet count, 13 positive end-expiratory pressure, 14 extracorporeal cardiopulmonary resuscitation, 15 CO₂ removal rate, 16 lactate, 17 secondary infection, 18 PaO2, 19 thrombocytopenia or low platelets, 20 age, 21 hypoglycemia, 22 diabetes mellitus, 23 fibrinogen, 24 kidney injury, 25 aortic cannulation, 26 pre-ECMO cardiac surgery, 27 ECMO blood flow rate, 28 International Normalized Ratio (INR), 29 activated partial thromboplastin time (aPTT), 30 oxygenation index, 31 non-white, 32 vasoactive drug, 33 pneumonia, 34 cerebral oxygen saturation, 35 IABP (Intra-aortic balloon pump) support, 36 Serum, 37 low pulse pressure, 38 obesity, 39 urgency of the index procedure.

### 3.3 Quality assessment score

Among the included studies, 22 were rated as high quality and 7 as moderate quality in the quality assessment. The results of the quality assessment were presented in Table 1.

### 3.4 Meta-analysis

The meta-analysis identified female sex (OR = 0.82, 95%CI: 0.69–0.98), pre-ECMO cardiac arrest (OR = 2.53, 95%CI: 2.06–3.10), CRRT (OR = 1.43, 95%CI: 1.08–1.90), and cardiac dysfunction (OR = 1.72, 95%CI: 1.15–2.58) as influencing factors of composite neurological complicaitons in adult ECMO patients (see Table 2). A leave-one-out sensitivity analysis was performed to explore the source of the high heterogeneity observed for CRRT. We found that exclusion of one study by Shi et al. (10) reduced the I^2^ statistic from 88.8 to 0.0%, indicating that this study was the primary contributor to the observed heterogeneity.

**Table 2:** Factors associated with composite neurological complications in adult ECMO patients.

Factors	Effect model	Heterogeneity Test		Pooled effect size (OR, 95%CI)	*Z*	*p-*value
		*I^2^* statistic	*p-*value			
Female sex	Fixed	0.0%	0.42	0.82, 0.69–0.98	2.22	0.03
Pre-ECMO cardiac arrest	Fixed	5.1%	0.35	2.53, 2.06–3.10	8.92	<0.001
CRRT	Random	88.8%	<0.001	1.43, 1.08–1.90	2.48	0.01
Cardiac dysfunction	Random	63.5%	0.03	1.72, 1.15–2.58	2.65	0.01

Female sex (OR = 1.66, 95%CI: 1.05–2.60) and ECMO duration (OR = 1.02, 95%CI: 1.00–1.04) as independent factors were significantly associated with stroke in adult ECMO patients; see Table 3.

**Table 3:** Factors associated with stroke in adult ECMO patients.

Factors	Effect model	Heterogeneity test		Pooled effect size (OR, 95%CI)	*Z*	*p-*value
		*I^2^* statistic	*p-*value			
Female sex	Fixed	17.7%	0.30	1.66, 1.05–2.60	2.19	0.03
ECMO duration	Fixed	0.0%	0.83	1.02, 1.00–1.04	1.97	0.05

Female sex (OR = 1.94, 95%CI: 1.15–3.27), pre-ECMO lactate (OR = 1.05, 95%CI: 1.01–1.10), pre-ECMO pH (OR = 0.03, 95%CI: 0.00–0.32), platelet count (OR = 0.92, 95%CI: 0.86–0.99), low platelets (OR = 2.68, 95%CI: 1.01–7.09), aPTT (OR = 1.10, 95%CI: 1.01–1.21), and vasoactive drug(OR = 1.73, 95%CI: 1.21–2.46) were risk factors of intracranial hemorrhage in adult ECMO patients; see Table 4. A leave-one-out sensitivity analysis was performed to explore the source of the high heterogeneity observed for low platelets. We found that exclusion of one study by Yu et al. (11) reduced the I^2^ statistic from 85.4 to 31.4%, indicating that this study was the primary contributor to the observed heterogeneity.

**Table 4:** Factors associated with intracranial hemorrhage in adult ECMO patients.

Factors	Effect model	Heterogeneity test		Pooled effect size (OR, 95%CI)	*Z*	*p-*value
		*I^2^* statistic	*p-*value			
Female sex	Random	63.1%	0.04	1.94, 1.15–3.27	2.49	0.01
Pre-ECMO lactate	Fixed	0.0%	0.55	1.05, 1.01–1.10	2.36	0.02
Pre-ECMO pH	Fixed	0.0%	0.99	0.03, 0.00–0.32	2.88	0.01
Platelet count	Random	78.4%	0.01	0.92, 0.86–0.99	2.02	0.04
Low platelets	Random	85.4%	<0.001	2.68, 1.01–7.09	1.98	0.05
aPTT	Fixed	0.0%	0.72	1.10, 1.01–1.21	2.12	0.03
Vasoactive drug	Fixed	0.0%	0.95	1.73, 1.21–2.46	3.02	0.00

### 3.5 Sensitivity analysis

Sensitivity analysis was performed by switching the effect models (fixed-effects vs. random-effects) for statistically significant factors. The results demonstrated that female sex in the stroke group became statistically non-significant after model conversion, indicating a lack of robustness. Other factors remained statistically significant with stable effect sizes, confirming their robustness. Detailed results are presented in Table 5.

**Table 5:** Sensitivity analysis results.

Factors	Original effect model (OR, 95%CI)	Transformed effect model (OR, 95%CI)
Female sex	1.66, 1.05–2.60	1.63, 0.96–2.78

### 3.6 Publication bias test

**Figure 3:**
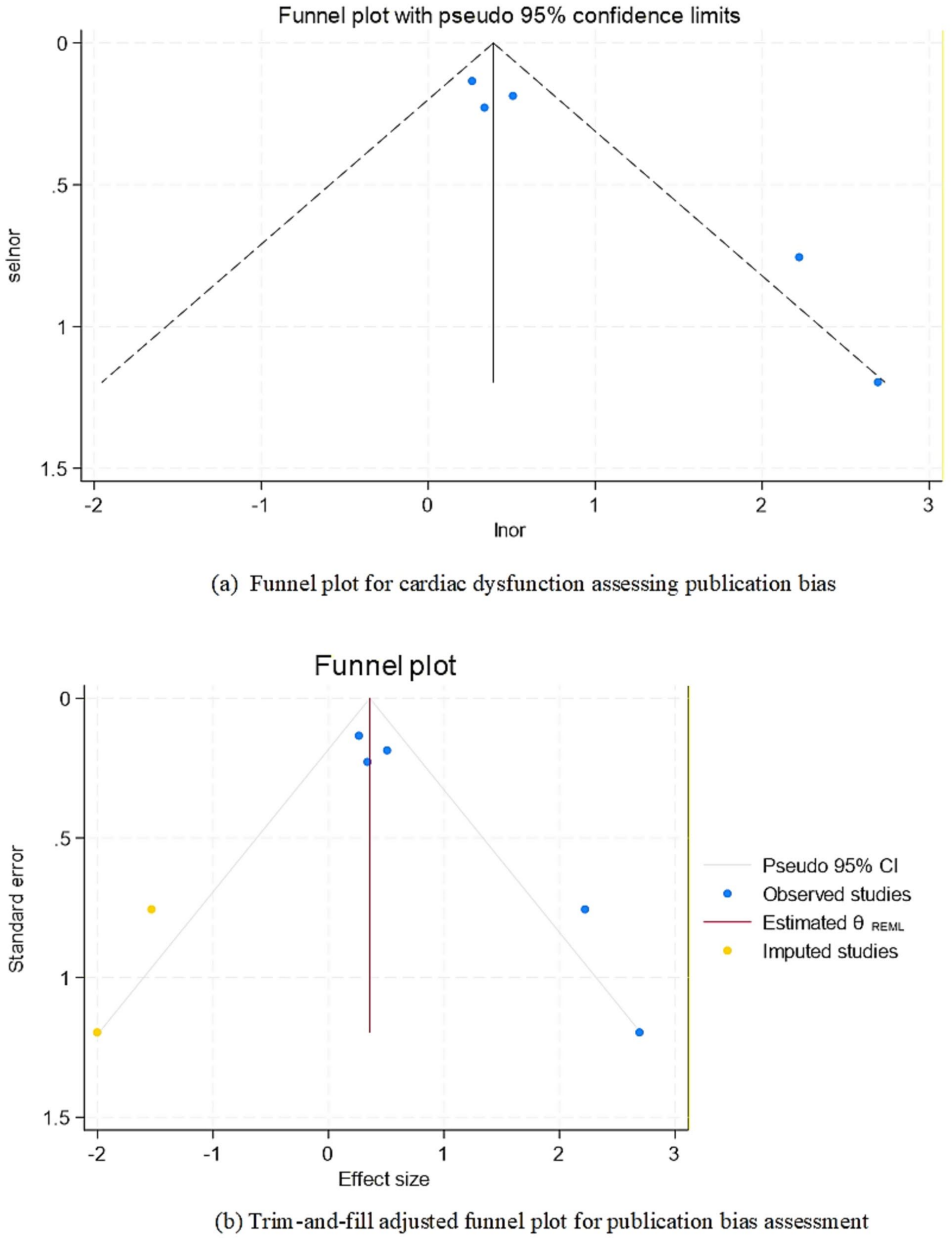
Funnel plot for publication bias: **(a)** Funnel plot for cardiac dysfunction assessing publication bias f. Funnel plot of the association between lnor and selnor. Each circle represents an individual study, with size proportional to its weight in the meta-analysis. The solid dashed line indicates the pooled odds ratio (OR = 1.72, 95% CI: 1.15–2.58), and the diagonal lines show the expected 95% confidence intervals under the null hypothesis of no bias. Asymmetry in the plot (Egger’s test, *p* = 0.02) suggests potential publication bias. **(b)** Trim-and-fill adjusted funnel plot for publication bias assessment f. Funnel plot of the association between or and se, adjusted for potential publication bias using the trim-and-fill method. Blue color circles denote the original studies (*n* = 5), while yellow color circles represent the 2 imputed studies added to restore symmetry. The red color dashed line shows the adjusted estimate after imputation (OR = 1.31, 95% CI: 1.02–1.68). The trim-and-fill analysis suggested left-side asymmetry, implying potential under-reporting of small negative studies.

Among the factors influencing composite neurological complications in adult ECMO patients, Egger’s test for cardiac dysfunction showed significant publication bias (*p* = 0.02 < 0.05). The trim-and-fill method imputed 2 missing studies, and the adjusted pooled effect size remained statistically significant (OR = 1.31, 95%CI: 1.02–1.68, *p* < 0.05), indicating robust results. Furthermore, the funnel plot after trim-and-fill adjustment showed no substantial asymmetry, suggesting no significant publication bias (Figure 3).

For stroke risk factors, the Egger’s test for ECMO duration showed no significant publication bias (*p* = 0.31). For intracranial hemorrhage risk factors, the funnel plot for female sex demonstrated symmetrical scatter distribution within the funnel boundaries, and Egger’s test confirmed no significant publication bias (*p* = 0.22).

### 3.7 Descriptive analysis

Since multiple influencing factors were reported in only single studies or had data unsuitable for pooling, a descriptive analysis was performed. For example, CO₂ removal rate (12), cerebral oxygen saturation (13), INR (14), fibrinogen (15), ECMO blood flow rate (14), oxygenation index (16), post-ECMO hypoglycemia (17) were all identified as potential risk factors.

## 4 Discussion

This meta-analysis identified 4, 2 and 7 influencing factors for composite neurological complications, stroke, and intracranial hemorrhage, respectively. The findings provide a comprehensive characterization of risk factors, which may facilitate the development of predictive models to identify high-risk patients. These results could serve as a valuable reference for improving overall outcomes in ECMO patients.

We have condensed these factors into two overarching categories: intrinsic risk factors and modifiable risk factors. Female sex was classified as an intrinsic factor, and we emphasize that clinicians should maintain heightened vigilance regarding potential neurological outcomes in this population. In contrast, factors such as the internal homeostasis, ECMO duration, coagulation parameters, cardiovascular stability, and other treatment-related parameters were categorized as modifiable risk factors. These elements can be optimized through clinical interventions, and their early identification and targeted management may help reduce the likelihood of adverse neurological outcomes.

### 4.1 Intrinsic factors—female sex

The findings of this study have identified female sex as a significant influencing factor. Notably, the direction of the pooled effect estimate for composite neurological complications differed from that of stroke and intracranial hemorrhage, which may be attributable to differences in sample size and statistical weighting among the included studies. In females, estrogen is thought to influence thrombin activity by enhancing procoagulant pathways through upregulation of clotting factors and suppression of anticoagulant proteins (18). During ECMO support, the requirement for systemic anticoagulation may further challenge the coagulation–fibrinolytic equilibrium, potentially increasing the risk of bleeding in female patients. On the other hand, studies have shown that estrogen receptors play a dominant role in platelet circulation, and estrogen may reduce platelet aggregation (19), further increasing the risk of bleeding in females. In addition, a cohort study found that, under the same dose of heparin, female patients undergoing elective surgery had significantly longer activated clotting times (ACT) and a higher incidence of bleeding complications compared to males (20). This suggests that females may achieve higher anticoagulation levels with the same heparin dose, indicating a potentially greater sensitivity to anticoagulant therapy. This could be attributed to lower average body weight in females or sex-related differences in pharmacokinetics. Therefore, anticoagulation management during ECMO support in female patients should be strictly regulated. Closer monitoring protocols are warranted, with particular attention to laboratory parameters and signs of bleeding tendency. In the future, anticoagulant dosing strategies may need to take into account sex-specific factors such as differences in body weight and variations in endogenous estrogen levels.

### 4.2 Modifiable risk factors—ECMO support therapy characteristics

#### 4.2.1 Internal homeostasis

The pre-ECMO pH and pre-ECMO lactate were risk factors for intracranial hemorrhage. Acid–base homeostasis is vital for sustaining life, and a decrease in pH combined with elevated lactate levels indicates a risk of intracellular acidosis. Acidosis can directly impair neuronal function by disrupting cellular energy metabolism and promoting intracellular accumulation of sodium and calcium ions (21), thereby increasing the risk. On the other hand, a drop in pH leads to physiological cerebral vasodilation, which increases cerebral blood flow and perfusion (22). However, in cases of severe acidosis, abnormal vasodilation may result in elevated intracranial pressure, potentially causing brain herniation, thereby aggravating ischemic and hypoxic injury to brain tissue. Moreover, elevated lactate levels have also been shown to exert neurotoxic effects. Excessive lactate enhances neuronal susceptibility to ischemic injury, thereby increasing the risk of ischemic brain damage (23).

#### 4.2.2 ECMO duration

The results indicated that prolonged ECMO support was a risk factor for stroke. Specifically, each additional day of ECMO support was associated with a 2% increase in stroke risk, which was consistent with previous research. Studies have shown that even a one-day extension in ECMO duration was associated with increased risks of complications such as bleeding events and renal failure (24). Other research had reported that, compared with patients without neurological complications, those who developed neurological complications exhibited significantly elevated lactate levels 24 h after ECMO initiation (25). An increased number of complications and elevated lactate levels might further impair normal brain function and increase the risk of neurological injury.

### 4.3 Modifiable risk factors—treatment-related characteristics

#### 4.3.1 Cardiovascular stability

Meta-analysis results indicated that cardiac dysfunction and cardiac arrest were independent risk factors for composite neurological complication. In patients with cardiac insufficiency, the significantly reduced cardiac output may lead to decreased cerebral blood flow and impaired cerebral perfusion. The resulting inability to maintain effective perfusion within brain tissue contributes to heterogeneous cerebral microcirculation (26), thereby increasing the risk of neurological complications. Moreover, during cardiac arrest, cerebral blood flow is completely interrupted. Although timely chest compressions can provide partial blood supply to the brain, prolonged inadequate cerebral perfusion can lead to severe cerebral ischemia and hypoxia, subsequently resulting in neurological complications. On the other hand, from the perspective of VA-ECMO support itself, the retrograde blood flow into the aorta increases left ventricular afterload, and elevates end-diastolic pressure, which may further increase the risk of pulmonary edema, cerebral ischemia and hypoxia (27).

We found that vasoactive drug use correlated with intracranial hemorrhage. Moreover, blood pressure fluctuations significantly impact the cerebrovascular system. Studies have shown that in the setting of hypertension, reactive oxygen species generated by oxidative stress can directly or indirectly damage normal structures such as cerebrovascular endothelial cells and neurons, leading to cerebrovascular disease and ultimately resulting in cerebral ischemia and hypoxia (28). This highlights the significant role of elevated blood pressure in the development of cerebrovascular disorders, which is consistent with the results of this study.

#### 4.3.2 Coagulation parameters

Thrombocytopenia, decreased platelet count, and prolonged APTT are risk factors for intracranial hemorrhage in ECMO patients. This conclusion aligns with multiple previous studies. Under normal circumstances, after vascular injury, platelets adhere to the vascular endothelium and aggregate to form platelet plugs, thereby achieving hemostasis (29). However, when the platelet count is too low, effective platelet plug formation is impaired, leading to hemorrhage. Moreover, the systemic anticoagulation strategy during ECMO support further suppresses coagulation function, making microbleeds prone to progress into intracranial hemorrhage. Prolonged APTT indicates dysfunction of the intrinsic coagulation pathway, reduced thrombin generation, and impaired fibrin clot formation, thereby increasing spontaneous bleeding tendency and making multi-site cerebral hemorrhage more likely. This highlights the critical importance of optimizing anticoagulation management during ECMO support. Coagulation function should be dynamically monitored, with timely adjustments to the anticoagulation regimen. Platelet counts should be maintained within the normal range as much as possible, and proactive measures should be taken to prevent bleeding.

#### 4.3.3 Continuous renal replacement therapy status

This study also found that CRRT has a significant impact on the neurological system of patients receiving ECMO support. On the one hand, fluid overload is common in ECMO patients, and the combination of CRRT enables continuous and controlled fluid removal. However, rapid fluid shifts or episodes of hypotension during this process may lead to inadequate cerebral perfusion, resulting in ischemic brain injury. On the other hand, electrolyte disturbances and acid–base imbalances are frequent indications for CRRT. Rapid osmotic shifts caused can induce cerebral edema, thereby increasing the risk of neurological complications. In addition, systemic anticoagulation strategies—such as heparin or citrate—are routinely used during CRRT treatment (30). It is essential to closely monitor coagulation parameters and maintain a delicate balance between bleeding and thrombosis. Disruption of this balance may predispose patients to cerebral hemorrhage or stroke.

Given the wide temporal span of the included studies, we performed a sensitivity analysis using 2005 as the cut-off year, excluding earlier studies, to explore whether historical evidence might influence the association between ECMO and neurological complications in contemporary practice. We found that the early study by Ba et al. modified the identified risk factor female sex for composite neurological complications in modern ECMO cohorts, and that the early study by Ka et al. altered the identified risk factor low platelets for intracranial hemorrhage. In contrast, the remaining risk estimates were largely consistent after exclusion of early studies, suggesting that most associations are robust across eras.

Another important limitation of our study is the heterogeneous classification of ECMO support. VV ECMO, VA ECMO, and ECPR are applied to fundamentally different patient populations, with distinct underlying pathophysiology, indications, and severity of illness, all of which may influence neurological risk. However, in our dataset, ECMO mode was incompletely reported, and several contributing studies labeled support only as “ECMO” without further distinction. As a consequence, we were unable to systematically stratify outcomes according to ECMO mode or to perform robust mode-specific analyses. The results presented here therefore reflect an aggregate effect across mixed ECMO strategies and should be interpreted as hypothesis-generating rather than definitive with respect to any individual mode. Future large-scale, prospective, and preferably multicenter studies using standardized definitions of neurological complications, contemporary ECMO configurations, and detailed information on ECMO mode and indication are needed to confirm these observations and to refine risk stratification in present-day ECMO populations.

## 5 Conclusion

This study conducted a systematic review and meta-analysis of factors influencing neurological complications in adult ECMO patients, identifying that female sex, comorbid illnesses, pre-ECMO internal homeostasis characteristics, ECMO support therapy-related characteristics, and anticoagulation characteristics all contribute to neurological complications. Based on these risk factors, healthcare providers should prioritize neurological complications prevention. Future research may develop risk prediction models to facilitate early identification of high-risk patients and formulate targeted intervention strategies, thereby improving overall outcomes in ECMO patients.

## 6 Strengths and limitations

The main strengths of this study include: (1) a comprehensive search strategy was implemented, covering both Chinese and English databases, supplemented by gray literature retrieval and manual reference tracing to ensure completeness of the evidence base; (2) the entire process was conducted in strict accordance with the PRISMA guidelines, ensuring methodological rigor and transparency; (3) the findings provide a degree of novelty by offering updated and integrated evidence on the association between female sex and neurological complications.

The limitations include: (1) the limited number of original studies precluded subgroup analyses, restricting the ability to explore differences across specific study characteristics; (2) substantial heterogeneity was observed in some pooled estimates, which may affect the stability and precision of the overall results; (3) for certain covariates, fewer than 10 studies were available, leading to insufficient statistical power for the Egger test and a potential risk of publication bias; (4) the robustness of the association between female sex and stroke was limited, which may be influenced by residual confounding, study heterogeneity, and the relatively small number of included studies; (5) all included studies were observational in nature, making it difficult to fully control for unmeasured confounders and inherently limiting causal inference; (6) moreover, the inclusion of several small-sample studies and the inability to perform more granular subgroup analyses at this level may introduce additional confounding, and future large-scale, multicenter studies are warranted to validate our findings.

These limitations highlight the need for future high-quality, large-scale studies to further validate our findings.
